# Winter cover crop suppression methods influence on sunflower growth and rhizosphere communities

**DOI:** 10.3389/fmicb.2024.1405842

**Published:** 2024-06-26

**Authors:** Marianela Estefanía Morales, Marco Allegrini, Jessica Basualdo, Gastón Alejandro Iocoli, María Bonita Villamil, María Celina Zabaloy

**Affiliations:** ^1^Centro de Recursos Naturales Renovables de la Zona Semiárida (CERZOS), Universidad Nacional del Sur (UNS)-CONICET, Bahía Blanca, Argentina; ^2^Departamento de Agronomía, Universidad Nacional del Sur, Bahía Blanca, Argentina; ^3^Department of Crop Sciences, University of Illinois, Urbana, IL, United States

**Keywords:** winter cover crop, sunflower growth, rhizospheric microorganisms, rolling, glyphosate

## Abstract

Sunflower (*Helianthus annuus* L.), a vital crop for global vegetable oil production, encounters sustainability challenges in its cultivation. This study assesses the effects of incorporating a winter cover crop (CC), *Avena sativa* (L.), on the subsequent growth of sunflower crops and the vitality of their rhizosphere microbial communities over a two-year period. It examines the impact of two methods for suppressing winter CC—chemical suppression using glyphosate and mechanical suppression via rolling—both with and without the addition of phosphorus (P) starter fertilizer. These approaches are evaluated in comparison to the regional best management practices for sunflower cultivation, which involve a preparatory chemical fallow period and the subsequent application of starter P fertilizer. The methodology utilized Illumina sequencing for the analysis of rhizosphere bacterial 16S rRNA genes and fungal internal transcribed spacer (ITS) amplicons. Findings indicate a significant improvement (9–37%) in sunflower growth parameters (plant height, stem diameter, head diameter, and head dry weight) when cultivated after glyphosate-suppressed winter CC compared to the chemical fallows. Conversely, rolling of winter CC generally negatively affected sunflower growth. Rhizosphere bacterial communities following chemical suppression of winter CC showed greater Pielou’s evenness, indicating a uniform distribution of species. In general, this treatment had more detrimental effects on beneficial sunflower rhizosphere bacteria such as *Hymenobacter* and *Pseudarthrobacter* than rolling of the winter CC, suggesting that the overall effect on sunflower growth may be mitigated by the redundancy within the bacterial community. As for fungal diversity, measured by the Chao-1 index, it increased in sunflowers planted after winter CC and receiving P fertilization, underscoring nutrient management’s role in microbial community structure. Significant positive correlations between fungal diversity and sunflower growth parameters at the reproductive stage were observed (*r* = 0.41–0.72; *p* < 0.05), highlighting the role of fungal communities in plant fitness. The study underscores the positive effects of winter CC inclusion and management for enhancing sunflower cultivation while promoting beneficial microbes in the crop’s rhizosphere. We advocate for strategic winter CC species selection, optimization of mechanical suppression techniques, and tailored phosphorus fertilization of sunflower to foster sustainable agriculture.

## 1 Introduction

Sunflower (*Helianthus annuus* L.) stands as a globally significant crop for vegetable oil production, ranking behind only soybean (*Glycine max* L. Merr.), rapeseed (*Brassica napus* L.), and palm (*Elaeis guineensis* Jacq.) in terms of cultivation area and oil yield. Argentina, as a leading figure among the world’s top sunflower oil producers, boasts an extensive potential cultivation area stretching from the Chaco region in the north to the Pampas in the south. In the 2022/23 season, sunflower crops were sown over 2.2 million hectares, with 75% of this area employing no-tillage farming methods (RETAA, 2023), a practice that, while reducing labor costs and enhancing soil properties (Díaz-Zorita et al., 2002), has led to an increased reliance on glyphosate for weed management. This dependence has, in turn, contributed to the emergence of glyphosate-resistant weed species (Castaño, 2018; Osipitan et al., 2018).

Amid these challenges, winter cover crops (CC) have emerged as a promising strategy to mitigate some of the issues associated with no-tillage systems, such as soil erosion, organic matter depletion, and the rise in herbicide resistance. Winter CC are recognized for their potential to improve soil health, conserve moisture, and suppress diseases by enhancing the activity of beneficial microbial communities, including plant growth-promoting bacteria, and fungal antagonists (Agaras et al., 2015; Osipitan et al., 2018; Adetunji et al., 2020; Garba et al., 2022). Despite these advantages, the impact of winter CC on the yields of subsequent primary crops, such as sunflower, has shown variability, with some studies reporting no significant benefits or even yield penalties when compared to traditional fallow practices (Rosner et al., 2018; de Sá Pereira et al., 2020). In the semi-arid region of the southwest of Buenos Aires Province, winter cereal crops such of wheat (*Triticum aestivum* L.) and barley (*Hordeum vulgare* L.) are typically grown in monoculture, rotated with sunflower, or with a year-long fallow in between crops (Venanzi et al., 2004). Average sunflower yield is 500 kg ha^−1^, mostly constrained by water availability. Conservation practices are implemented only on approximately 15% of the area (Schmidt et al., 2018). In this area, the use of cover crops has been limited due to the potential consumptive water use by winter CC at the expense of subsequent crop cultivation. However, in dry years or even in years with average annual rainfall, some field studies have shown that it is possible to include winter CC in rotations with low-density sunflower or corn to maintain soil health and protection without affecting water availability (De Leo et al., 2020; de Sá Pereira et al., 2020).

The necessity of winter CC suppression before planting primary crops introduces additional complexity. Methods including glyphosate use or mechanical means such as rolling or cutting can influence the efficacy of winter CC in improving subsequent crop yields. While chemical suppression is widely used for its effectiveness and convenience, concerns have been raised about glyphosate’s persistence in the soil and its potential impact on non-target crops and soil health (Kremer et al., 2005; Al-Rajab et al., 2008; Bott et al., 2011; Duke et al., 2012). This scenario underscores the importance of exploring alternative suppression methods that minimize negative impacts on soil health and crop performance.

Recent advances in metagenomics and high-throughput sequencing technologies have revolutionized the ability to study the rhizosphere microbiome, offering insights into the complex interactions between plants and their microbial communities. These technologies enable a deeper understanding of how winter CC management practices, including suppression methods and the application of phosphorus fertilization, influence the structure and function of rhizosphere microbial communities (Philippot et al., 2013; Kumar and Dubey, 2020; Liu et al., 2021). Recognizing these benefits and challenges, recent research (Villamil et al., 2021; Kim et al., 2022; Morales et al., 2022) has delved deeper into the effects of CC management practices on soil microbial communities.

A foundational study conducted at the same experimental site provides pertinent insights into the nuances of winter CC management effects on soil microbiomes. Morales et al. (2022) assessed the impact of winter CC suppression methods on the oats (*Avena sativa* L.) rhizosphere microbiome, utilizing Illumina sequencing to analyze bacterial 16S rRNA gene and fungal ITS amplicons. Their investigation revealed that oats suppression methods selectively favored certain bacterial genera, including those acting as fungal antagonists and chitin degraders, while also identifying two fungi as potential soil-borne pathogens. Conversely, these suppression methods adversely impacted other genera known for their plant growth-promoting functions. Given the persistence of senescent roots from suppressed winter CC within the soil matrix, these findings underscore the imperative to further examine the effects of winter CC suppression methods on the rhizospheric microbiome of subsequent crops. This highlights a critical area of investigation for enhancing agricultural sustainability and soil health, framing the context for our study.

Building on these foundational insights, our study aims to assess the effects of a winter CC and its suppression methods on the growth and reproductive success of sunflower crops, as well as the composition and diversity of bacterial and fungal communities in the sunflower rhizosphere. We hypothesize that the method of suppressing the winter CC, whether through chemical means using glyphosate or mechanical means via rolling, in conjunction with or without the addition of phosphorus starter fertilizer, will influence sunflower growth and rhizosphere microbial communities differently compared to regional best management practices. By exploring these interactions, this research seeks to contribute valuable knowledge toward optimizing management practices for sunflower cultivation that support both environmental health and crop productivity.

## 2 Materials and methods

### 2.1 Characterization of the study area

The study was conducted over two years (2018 and 2019) at the Napostá Experimental Field, located in the Bahía Blanca district, southwest of the Buenos Aires province, Argentina (38°25′39”S, 62°17′41” W) (Supplementary Figure S1). This area lies within the middle portion of the Subventania plain, characterized by gently undulating terrain with dominant soils derived from loess deposited on an underlying petrocalcic horizon (Schmidt et al., 2018).

The climate is temperate semiarid with a mean annual temperature of 15°C and a mean annual rainfall recorded over the period from 1959 to 2014 of 654 mm, with approximately two-thirds of it occurring in autumn and spring. There is a dry season in late winter and a semi-dry season in mid-summer (January and February). In 2018, the mean annual precipitation was 580 mm, while in 2019, it was 506 mm.

Experimental plots were established on loamy Petrocalcic paleustoll soil (Ap-A2-AC-2Ck-3Ckm) as classified by the Soil Survey Staff, NRCS, and USDA (2019). The area had an average topsoil pH of 6.7 (1:2.5 soil:water), 4.24% soil organic matter determined by dry combustion with an automatic analyzer (LECO, St. Joseph, MI, USA), 8 mg kg^−1^ available P, 14.4 mg kg^−1^ of NH_4_^+^, and 10.6 mg kg^−1^ of NO_3_^−^ as available N forms.

### 2.2 Treatment and cultural practices

This study assesses sunflower management practices (SMP) by comparing four experimental strategies against the regionally endorsed best management practice for sunflower cultivation, which serves as the control treatment (CT). The standard approach for the control involves a chemical fallow period before the sunflower planting, accompanied with starter phosphorus (P) fertilizer during planting (Aguirrezábal et al., 1996). The experimental strategies introduce a winter cover crop (CC) of oats, which is subsequently suppressed before planting the sunflower crop. This suppression is achieved through either chemical means using glyphosate (DQ) or mechanical rolling (R). Following these treatments, the sunflower crop is either planted with (DQ/P, R/P) or without (DQ, R) the application of starter phosphorus fertilizer, exploring the impacts of these varied management practices on sunflower cultivation.

Each year, the treatments (SMP: 5 treatments) were arranged in randomized complete block design, with four replications per treatment. For all treatments but the control (CT), oats (*Avena sativa* L. var. Cristal INTA) were no-till seeded into crop residues in 15 cm rows, on each experimental unit (plots of 2.25 × 1.56 m) at a rate of 152 kg seeds ha^−1^ on June 3, 2018, and May 13, 2019. No herbicides were applied to any plot for the duration of the experiment, except as part of a treatment. Urea nitrogen (N) fertilizer (46% N) was surface applied in all plots at planting (40 kg N ha^−1^) of the CC. Oats growth was suppressed either chemically or mechanically at the Z55 stage (Zadoks et al., 1974). A commercial formulation of glyphosate (mixture of salts of N-[phosphonomethyl]glycine as active ingredient) was sprayed over control treatment and winter CC designated for chemical suppression (DQ and DQ/P) using a knapsack sprayer, while a roller-crimper was manually tracked over the oats designated for mechanical suppression (R and R/P) (Morales et al., 2022).

Sunflower, KWSol 480 CL (KWS Argentina S.A.) was planted at a density of 68,000 plants ha^−1^ within two weeks after the suppression of the CC in early November each year. Sunflower seeds were manually sown in 52 cm rows with a spacing of 45 cm in the same row. Phosphorous in the form of diammonium phosphate (DAP, 18–46-0) was applied in bands, 5 cm from the sowing line, at a rate of 30 kg ha^−1^ as part of the treatments DQ/P, R/P and CT. During the vegetative stage (V10, ten leaves), all plots received broadcast N fertilizer at a rate of 40 kg N ha^−1^ (Urea, 46% N). Supplementary irrigation was applied to all plots at planting and during the reproductive stage of sunflower (R1, Schneiter and Miller, 1981; Supplementary Table S1).

### 2.3 Collection of sunflower plant growth metrics and rhizosphere soil

Sunflower plant growth metrics were evaluated at two key growth stages annually: the vegetative stage (V10, ten leaves) and the reproductive stage (R6, complete anthesis).

During the vegetative stage, 2–3 plants per plot were measured for plant height (PHV), number of leaves (NLV), and harvested to determine dry weight of stems and leaves (DWSL). At the reproductive stage, all plants except those at the edges were measured and harvested. Measurements included plant height (PHR6), number of leaves (NLR6), stem diameter (SD), head diameter (HD), and dry weight (HDW).

Leaf enumeration included only those longer than 4 cm. Plant height was measured from the soil base to the last leaf, while stem diameter was assessed 2 cm above ground level. Plant material was oven-dried at 70°C to constant weight for dry weight determinations.

Plants collected during the vegetative stage were kept in an ice cooler during transport to the laboratory. Roots from each plot were moderately shaken to remove the bulk soil. The rhizospheric soil was then collected through manual detachment using a sterile brush, following the method described by Allegrini et al. (2019). Samples were stored at −80°C until DNA extraction.

### 2.4 DNA extraction and metagenomics analysis

DNA was extracted from rhizospheric soil samples using the DNeasy PowerSoil Kit (Qiagen, Hilden, Germany), according to the manufacturer’s protocol. A precise quantity of 250 mg of soil was used for each extraction to ensure consistency across samples. The concentration and purity of the extracted DNA were determined using the QuantiFluor® dsDNA System (Promega®, Madison, WI, USA) and verified through absorbance measurements at 260:230 and 260:280 nm ratios using a DS-11 FX Spectrophotometer (DeNovix Inc., Wilmington, DE, USA). Quality was further assessed by agarose gel electrophoresis to check for DNA integrity.

Amplicon sequencing was conducted using the three biological replicates with the best quality extracted DNA out of the four replicates available per treatment for the first year sample set. In the second year, all four replicates were included in the sequencing regardless of their quality to capture most of the variability arising from harsh climatic conditions. The V4 region of the bacterial 16S rRNA gene and the fungal internal transcribed spacer (ITS) region were sequenced using the MiSeq platform (Illumina, Inc., San Diego, CA, USA) with a paired-end approach (2 × 300 bp). Amplification of the bacterial 16S rRNA gene utilized primers 515F and 806R, while fungal ITS regions were amplified with primers 7F and 4R (Supplementary Table S2), following the Fluidigm™ protocol at the Roy J. Carver Biotechnology Center, University of Illinois at Urbana-Champaign, USA.

### 2.5 Bioinformatics analysis of the microbial community

Sequence processing was performed in QIIME2 (Bolyen et al., 2019), utilizing the DADA2 algorithm (Callahan et al., 2016) for quality control steps such as primer removal, trimming, and chimera filtering. This resulted in high-quality amplicon sequence variants (ASVs). Trimming parameters were optimized to 283 bp for forward reads and 251 bp for reverse reads for bacteria, and 294 bp for forward and 280 bp for reverse reads for fungi, based on QIIME2’s Interactive Quality Plot tool, with an expected error threshold set to 2.

Extracted ASVs underwent taxonomic classification using the Ribosomal Database Project (RDP) Classifier v2.11 (Wang et al., 2007). Diversity within samples (alpha-diversity) was quantified through metrics such as the observed number of ASVs, Chao-1 index, Shannon diversity index, reciprocal Simpson index, and Pielou’s evenness, as per Allegrini et al. (2019). These analyses provided insights into the richness and evenness of microbial communities.

For phylogenetic analyses, ASVs sequences were aligned using MAFFT v7 (Katoh et al., 2019), and a neighbor-joining tree was constructed with the phangorn package v2.5.5 (Schliep, 2011) in R. This tree was instrumental in calculating generalized UniFrac distances for assessing beta diversity, leveraging the GUniFrac package v1.1 (Chen et al., 2022). Community composition differences between treatments were explored via non-metric multidimensional scaling (NMDS) and nonparametric multivariate analysis of variance (NPMANOVA) with 1,000 permutations, using the vegan package v2.5–6 (Oksanen et al., 2022). Treatment effects on microbial structure were further analyzed through pairwise PERMANOVA tests, adjusting for false discovery rates (“FDR”) with the RVAideMemoire package v0.9–80 (Hervé, 2022).

### 2.6 Statistical analysis

The statistical analysis began with the initial dataset of amplicon sequence variants (ASVs), derived from the Ribosomal Database Project (RDP) classifier, encompassing 18,896 bacterial ASVs and 2,463 fungal ASVs. The first step involved aggregating ASVs reads by genera, leading to 1,157 bacterial and 380 fungal genera. These were further refined to include only genera with average relative abundances greater than 0.1%, as per the methodology outlined by Gloor and Reid (2016). This filtration resulted in 188 bacterial and 121 fungal genera.

To identify the genera most responsive to treatment variations, a bootstrap forest partitioning method was used within the JMP® predictor screening platform (SAS Institute Inc, 2018; Villamil et al., 2021). This approach pinpointed 26 bacterial and 4 fungal genera as significant contributors (≥1% contribution) to model variability. Data were then transformed using the centered log-ratio (clr) method (Aitchison, 1982), with zero values addressed using the “cmultRep1” function from the zCompositions package in R (Palarea-Albaladejo and Martín-Fernández, 2015), aligning with recommendations for compositional data analysis (Gloor and Reid, 2016; Gloor et al., 2017).

Principal Component Analysis (PCA), executed via the FACTOR procedure in SAS v9.4 (SAS Institute, Cary, NC, USA), distilled bacterial genus abundances into principal components (PCs). PCs with eigenvalues ≥1, and accounting for at least 5% of dataset variability, were selected for further analysis. Genera strongly correlated with PCs (correlation coefficient > |0.45|) were identified as sensitive indicators for PCs descriptions (Villamil et al., 2021).

Linear mixed models were fitted using PROC GLIMMIX in SAS v9.4, to determine the effects of SMP treatments on sunflower growth parameters, alpha-diversity metrics, the fungal genera indicators, and the PCs scores of bacterial indicator genera. Treatments were considered fixed effects, whereas blocks, and the interactions with treatments were treated as random factors. Year was treated as a random effect and included as a repeated measure within the experimental units defined by block and treatment combination, with a variance components (VC) covariance structure to account for the variance attributable to different years (Littell et al., 2006; Stroup, 2012). Statistically significant differences among treatment means were determined using the least-square means separation, with a type I error (*α*) set at 0.05. Pearson’s correlation coefficients assessed relationships between plant variables, alpha-diversity, and bacterial PC scores, applying thresholds for “very strong” (>|0.8|), “strong” (|0.6–0.8|), and “moderate” (|0.4–0.6|) associations, following Kim et al. (2022).

**Figure 1:**
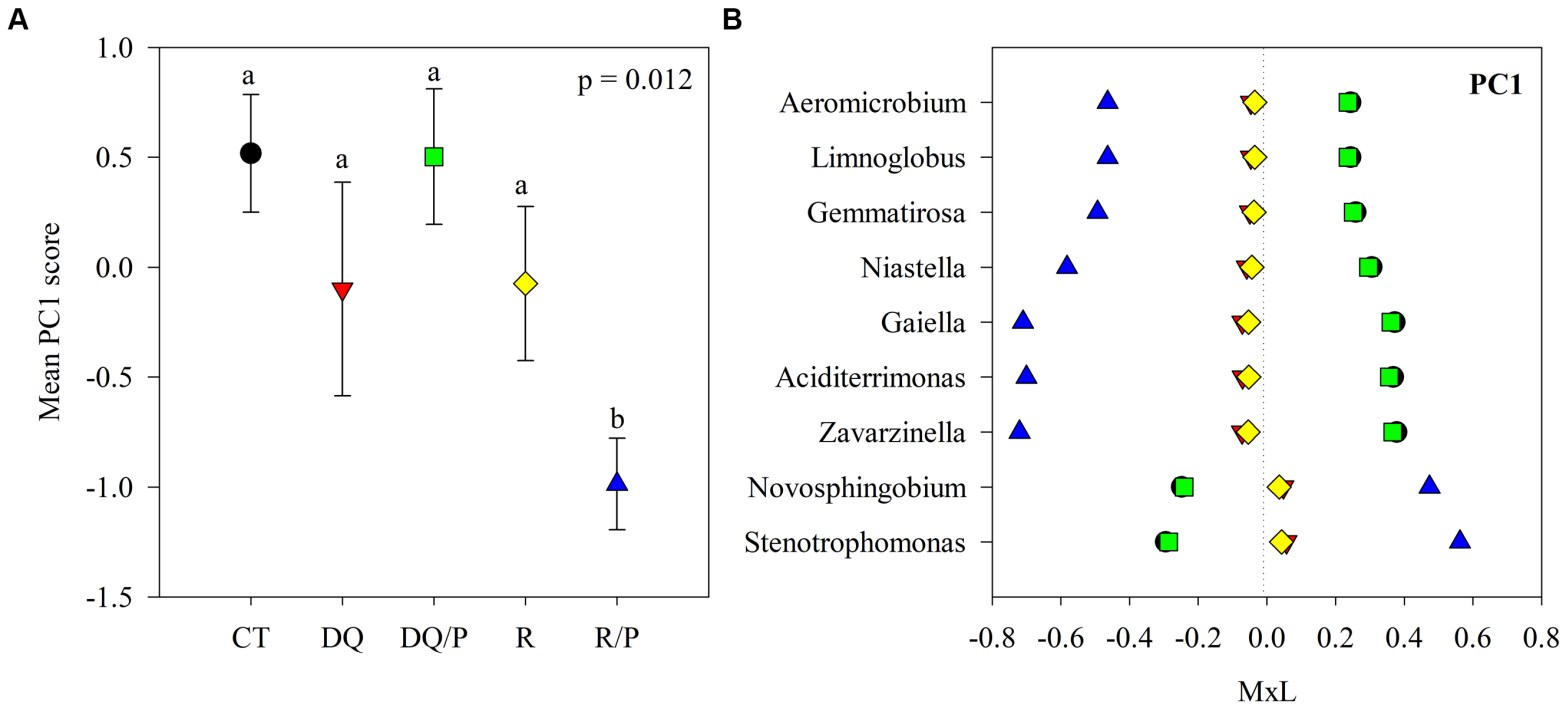
Results of the principal component analyses (PCAs) and their mean separation procedure showing the indicator bacterial genera according to Supplementary Table S4 for PC1. **(A)** Mean values of the PC1 scores for each treatment with their standard errors (as error bars). Different lower-case letters indicate significant differences between treatments (*α* = 0.05). **(B)** Contribution of each indicator genera to the PC1 mean value for each treatment (CT, black circles: control treatment; DQ, red inverted triangles: sunflower after winter CC chemical suppression without P at sowing; DQ/P, green squares: sunflower after winter CC chemical suppression with P fertilization at sowing; R, yellow rhombuses: sunflower after winter CC rolling without P at sowing; R/P, blue triangle: sunflower after winter CC rolling with P fertilization at sowing) multiplied by the loading of the specific genera within the PC (Supplementary Table S4), named M × L.

**Figure 2:**
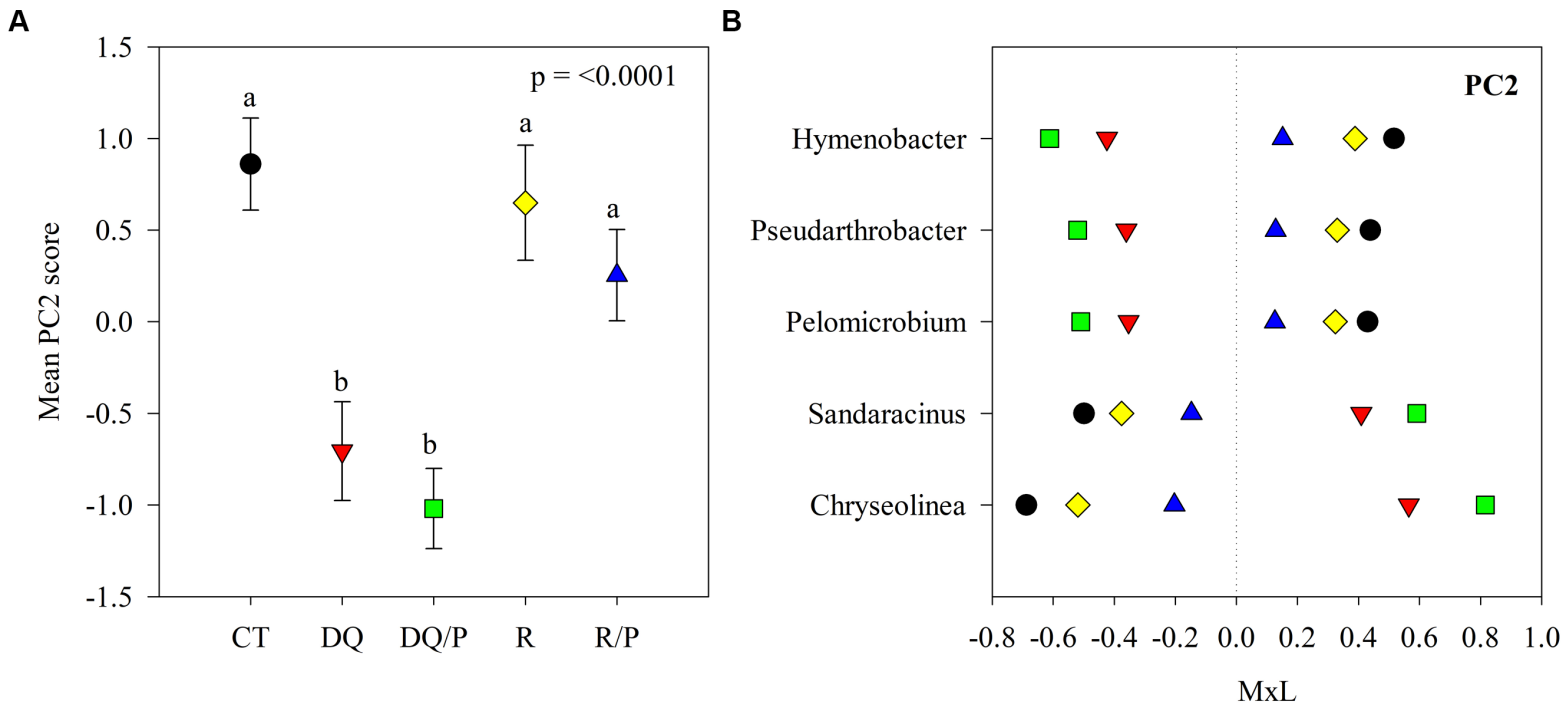
Results of the principal component analyses (PCAs) and their mean separation procedure showing the indicator bacterial genera according to Supplementary Table S4 for PC2. **(A)** Mean values of the PC2 scores for each treatment with their standard errors (as error bars). Different lower-case letters indicate significant differences between treatments (*α* = 0.05). **(B)** Contribution of each indicator genera to the PC2 mean value for each treatment (CT, black circles: control treatment; DQ, red inverted triangles: sunflower after winter CC chemical suppression without P at sowing; DQ/P, green squares: sunflower after winter CC chemical suppression with P fertilization at sowing; R, yellow rhombuses: sunflower after winter CC rolling without P at sowing; R/P, blue triangle: sunflower after winter CC rolling with P fertilization at sowing) multiplied by the loading of the specific genera within the PC (Supplementary Table S4), named M × L.

**Figure 3:**
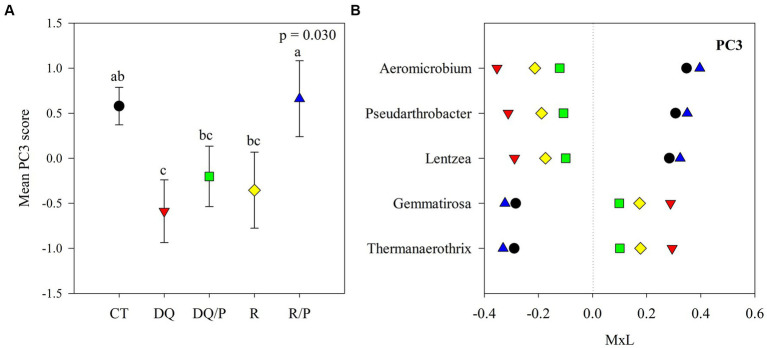
Results of the principal component analyses (PCAs) and their mean separation procedure showing the indicator bacterial genera according to Supplementary Table S4 for PC3. **(A)** Mean values of the PC3 scores for each treatment with their standard errors (as error bars). Different capital letters indicate significant differences between treatments (*α* = 0.05). **(B)** Contribution of each indicator genera to the PC3 mean value for each treatment (CT, black circles: control treatment; DQ, red inverted triangles: sunflower after winter CC chemical suppression without P at sowing; DQ/P, green squares: sunflower after winter CC chemical suppression with P fertilization at sowing; R, yellow rhombuses: sunflower after winter CC rolling without P at sowing; R/P, blue triangle: sunflower after winter CC rolling with P fertilization at sowing) multiplied by the loading of the specific genera within the PC (Supplementary Table S4), named M × L.

Graphical representations were produced with SigmaPlot v10.0 software. Each of Figures 1–3 comprises two panels. Panel (A) in each figure illustrates the mean PC score for each treatment, accompanied by their standard errors represented as whiskers. The second panel (B) displays the contribution of each indicator genus to the mean PC value for each treatment. This contribution is calculated by multiplying the mean PC value for a given treatment by the loading of the specific genus within the PC (Supplementary Table S4), denoted as M × L in each plot.

## 3 Results

### 3.1 Sunflower growth parameters

Table 1 presents the results of the analysis of sunflower growth parameters measured during the vegetative stage. It should be noted that one plot was lost from the R treatment in 2018, and one plot from the R/P treatment was lost in 2019. These losses were due to strong weed and cover crop competition, resulting in an uneven number of replicates (n) for these treatments. The results show highly significant differences for the variables number of leaves, plant height, and dry weight of stems and leaves (Table 1). Rolling (R) had the lowest number of leaves compared to control treatment and chemical suppression (DQ/P and DQ) (Table 1). Regarding plant height, control treatment and chemical suppression (DQ and DQ/P) had the highest values, while mechanical suppression (R and R/P) showed the lowest values (Table 1). Results observed for dry weight of stems and leaves, showed the highest values in control treatment, followed by chemical suppression (DQ and DQ/P), while the lowest values were recorded for mechanical suppression (R and R/P) (Table 1).

**Table 1:** Sunflower growth parameters measured in the vegetative stage.

		Number of leaves		Height of plant (cm)		Dry weight of stems and leaves (g)	
	*n*	Mean	SEM	Mean	SEM	Mean	SEM
Treatments^1^							
CT	8	10.36 A	1.14	16.65 B	2.99	6.80 A	1.12
DQ/P	8	9.74 A	1.14	20.03 A	2.99	2.94 B	1.12
DQ	8	9.92 A	1.14	21.11 A	2.99	3.58 B	1.12
R/P	7	3.85 B	1.14	9.15 C	3.01	0.25 C	1.16
R	7	3.53 B	1.14	7.76 C	3.01	0.40 C	1.16
	df	*p*-value					
Treatments	4	<0.0001		<0.0001		<0.0001	

Treatments mean values (mean), standard errors of the mean (SEM), number of observations (*n*), as well as probability values (*p*-value), and degrees of freedom (df) associated with the ANOVA of number of leaves, height of plant, and dry weight of stems and leaves. ^1^Treatments: CT, control treatment; DQ/P, sunflower after winter CC chemical suppression with P fertilization at sowing; DQ, sunflower after winter CC chemical suppression without P at sowing; R/P, sunflower after winter CC rolling with P fertilization at sowing; R, sunflower after winter CC rolling without P at sowing. Treatments mean values followed by different capital letters indicate statistical differences (*α* = 0.05).

The analysis of sunflower growth parameters measured at the reproductive stage (R6) (Table 2) also revealed significant differences for all variables, including plant height, number of leaves, stem diameter, head diameter, and head dry weight (Table 2). In the second year of the experiment, none of the plots in the rolled treatment (with or without P) reached the reproductive stage simultaneously with the strong competition with weeds and cover crops, along with severe drought conditions that affected the region later in the season. These factors led to the inability of plants in the rolled treatment to progress to the reproductive stage, making data collection at this stage incomplete for that treatment. The number of leaves in R6 was higher in the control treatment and chemical suppression (DQ and DQ/P) compared to mechanical suppression (R and R/P) (Table 2). For the variable plant height, chemical suppression (DQ and DQ/P) had the highest values, while control treatment and rolling (R) had higher values than the rolling with P treatment but did not differ among them (Table 2). The stem diameter showed the lowest values in mechanical suppression (R and R/P) (Table 2). Head diameter in control treatment was the smallest followed by rolling with P treatment, although they did not differ among them, while rolling (R) showed the highest values followed by chemical suppression (DQ and DQ/P) although the last ones did not differ between them nor from rolling with P (Table 2). The head dry weight variable in rolling with P showed the lowest values, followed by control treatment although they did not differ among them, while the highest values were recorded for chemical suppression (DQ and DQ/P) and rolling (R), although rolling did not differ from them nor control treatment (Table 2).

**Table 2:** Sunflower growth parameters measured in the reproductive stage.

		Number of leaves		Height of plant (cm)		Stem diameter (cm)		Head diameter (cm)		Head dry weight (g)	
	*n*	Mean	SEM	Mean	SEM	Mean	SEM	Mean	SEM	Mean	SEM
Treatments^1^											
CT	8	21.67 A	3.33	68.62 B	15.88	1.48 B	0.25	7.98 C	0.59	22.43 BC	3.65
DQ/P	8	22.94 A	3.33	85.74 A	15.88	1.57 AB	0.25	9.42 AB	0.59	29.23 A	3.65
DQ	8	23.29 A	3.33	88.07 A	15.88	1.67 A	0.25	9.45 AB	0.59	32.07 A	3.65
R/P	4	16.15 B	3.40	56.76 C	16.12	0.97 C	0.26	8.24 BC	0.72	18.01 C	4.61
R	3	17.40 B	3.43	70.38 B	16.25	1.20 C	0.27	9.92 A	0.77	28.81 AB	4.97
	df		*p*-value								
Treatments	4	<0.0001		<0.0001		<0.0001		0.007		0.009	

Treatments mean values (mean), standard errors of the mean (SEM), number of observations (*n*), as well as probability values (*p*-value), and degrees of freedom (df) associated with the ANOVA of number of leaves, height of plant, stem diameter, head diameter, and head dry weight. ^1^Treatments: CT, control treatment; DQ/P, sunflower after winter CC chemical suppression with P fertilization at sowing; DQ, sunflower after winter CC chemical suppression without P at sowing; R/P, sunflower after winter CC rolling with P fertilization at sowing; R, sunflower after winter CC rolling without P at sowing. Treatments mean values followed by different capital letters indicate statistical differences (*α* = 0.05).

### 3.2 Overall characterization of the sunflower rhizosphere microbial community

Metabarcoding analysis comprised 2,054,695 bacterial and 1,862,694 fungal sequences. After filtering, denoising, and removing chimeric sequences, the bacterial sequences were grouped into 18,896 ASVs, whereas the fungal sequences were grouped into 2,463 ASVs. The data have been deposited in NCBI Sequence Read Archive repository under the number accession PRJNA1034035.

The alpha-diversity measurements of observed richness (S′), reciprocal of Simpson index (1/λ), and Shannon index (H′), for bacteria and fungi, revealed no statistical differences among treatments (Table 3). On the other hand, the Pielou’s evenness index (J) for bacteria showed a statistically significant effect of the treatment (*p* = 0.013) with the highest value of J observed in chemical suppression with P and rolling, while the lowest J was recorded for both control treatment and rolling with P, and chemical suppression (DQ) exhibited intermediate values between these two groups. Treatments had a statistically significant effect on fungal Chao-1 index (*p* = 0.026). The diversity index under chemical suppression (DQ and DQ/P) and rolling with P was statistically higher than control treatment, with intermediate values for rolling (R) treatment.

**Table 3:** Treatment mean values (mean), standard errors of the mean (SEM), number of observations (*n*), as well as probability values (*p*-value), and degrees of freedom (df) associated with the ANOVA of the alpha-diversity metrics of Chao-1 index (estimated richness).

Taxa	Treatments^1^		Chao-1		S′		1/λ		H′		J	
		*n*	Mean	SEM	Mean	SEM	Mean	SEM	Mean	SEM	Mean	SEM
Bacteria	CT	7	1,241	80.58	1,236	80.05	558	41.48	6.73	0.07	0.945 B	0.004
	DQ/P	7	1,273	84.24	1,270	83.52	670	42.61	6.80	0.07	0.952 A	0.004
	DQ	7	1,234	80.58	1,231	80.05	600	41.48	6.72	0.07	0.949 AB	0.004
	R/P	6	1,262	85.43	1,260	84.71	624	42.71	6.77	0.07	0.947 B	0.004
	R	7	1,360	80.58	1,357	80.05	709	41.48	6.88	0.07	0.954 A	0.004
		df	*p*-value									
		4	0.764		0.755		0.120		0.474		0.013	
Fungi	Treatments^1^											
	CT	7	128.58 C	35.85	129.20	37.45	34.77	7.30	4.03	0.15	0.85	0.03
	DQ/P	7	158.19 AB	35.85	150.42	37.45	36.97	7.30	4.11	0.15	0.83	0.03
	DQ	7	164.16 AB	35.85	162.42	37.45	46.18	7.30	4.30	0.15	0.86	0.03
	R/P	6	178.12 A	36.04	177.44	37.51	53.54	7.62	4.46	0.16	0.87	0.03
	R	7	147.45 BC	35.85	146.01	37.45	45.93	7.30	4.25	0.15	0.87	0.03
		df	*p*-value									
		4	0.026		0.092		0.130		0.214		0.555	

S′, observed number of ASVs; 1/λ, reciprocal of Simpson index; H′, Shannon index; and J, Pielou’s evenness index. ^1^Treatments: CT, control treatment; DQ/P, sunflower after winter CC chemical suppression with P fertilization at sowing; DQ, sunflower after winter CC chemical suppression without P at sowing; R/P, sunflower after winter CC rolling with P fertilization at sowing; R, sunflower after winter CC rolling without P at sowing. Treatments mean values followed by different capital letters indicate statistical differences (*α* = 0.05).

The beta-diversity of bacteria and fungi (F. model = 1.38, p = 0.02 and F. model = 0.93, *p* = 0.553, respectively) indicated a significant treatment effect for bacteria only. However, pairwise comparisons (PERMANOVA) using the Benjamini and Hochberg correction (“FDR”) did not show significant differences (Supplementary Table S3). These results agreed with those of NMDS with generalized UniFrac distance where the separation between the bacterial and fungal communities of the sunflower rhizosphere was not observed as a result of treatments applied to the previous winter cover crop (Supplementary Figures S2A,B).

### 3.3 Compositional analysis of microbial communities in the sunflower rhizosphere

Supplementary Table S4 reports the results of the principal components (PCs) analysis that generated eight uncorrelated PCs with eigenvalue >1, which explained 71% of the variability in the data set. PC1 presented positive loadings (>0.45) for *Aciditerrimonas*, *Aeromicrobium*, *Gaiella*, *Gemmatirosa*, *Limnoglobus*, *Niastella,* and *Zavarzinella*, as well as negative loadings (<−0.45) for *Novosphingobium* and *Stenotrophomonas*. PC2 showed positive loadings for *Hymenobacter*, *Pelomicrobium,* and *Pseudarthrobacter* and negative loadings for *Chryseolinea* and *Sandaracinus*. PC3 had positive loadings for *Aeromicrobium*, *Lentzea,* and *Pseudarthrobacter* and negative loadings for *Gemmatirosa* and *Thermanaerothrix*. PC4 eigenvectors included positive loadings for *Arma*_gp5 and *Denitratisoma* and negative loadings for *Agromyces*. PC5 showed positive loading for *Pantoea* and negative loading for *Thermanaerothrix*. The PC6 eigenvector included negative loading for *Mucilaginibacter*. PC7 presented negative loading for *Denitratisome*. PC8 showed positive loading for *Chitinispirillum* and negative loading for *Limnoglobus*.

The eight PCs that were used as independent variables in the ANOVA, indicated statistically significant treatments effects on PC1, PC2, and PC3. No statistically significant effects were detected for PC4, PC5, PC6, PC7, or PC8 and thus, there will not be further discussed.

Figures 1A, 2A, 3A show plots of the PC1, PC2, and PC3 means for each treatment with the corresponding standard error bars and means separation results. The contribution of each bacterial genus to these results is showed in Figures 1B, 2B, 3B. Compared to rolling with P, the group of indicator microbes with positive loadings within PC1, significantly increased with chemical suppression (DQ and DQ/P), rolling, and control treatments, and the opposite behavior was observed for those indicators with negative loadings (Figure 1B). Within PC2, compared to control treatment and mechanical suppression (R and R/P), the two bacteria genera with negative loadings statistically increased in abundance with chemical suppression (DQ and DQ/P) treatment, while the opposite behavior was observed for three indicators with positive loadings (Figure 2B). For PC3 three bacteria with positive loading significantly decreased in the sunflower rhizosphere with chemical suppression (DQ and DQ/P) and rolling treatments, while the opposite response was observed for the bacterial genera with negative loading, with respect to control treatment and rolling with P (Figure 3B).

Only four genera of fungi were selected as indicators for the sunflower management practices evaluated in this study: *Alternaria, Arthrinium, Atradidymella*, and *Auricularia* spp. However, and likely due to the high level of variability encountered, no statistically significant effects of the treatments were detected when these genera were used as independent variables in the analysis of variance (Supplementary Table S5).

### 3.4 Pearson’s correlation matrix among variables

**Figure 4:**
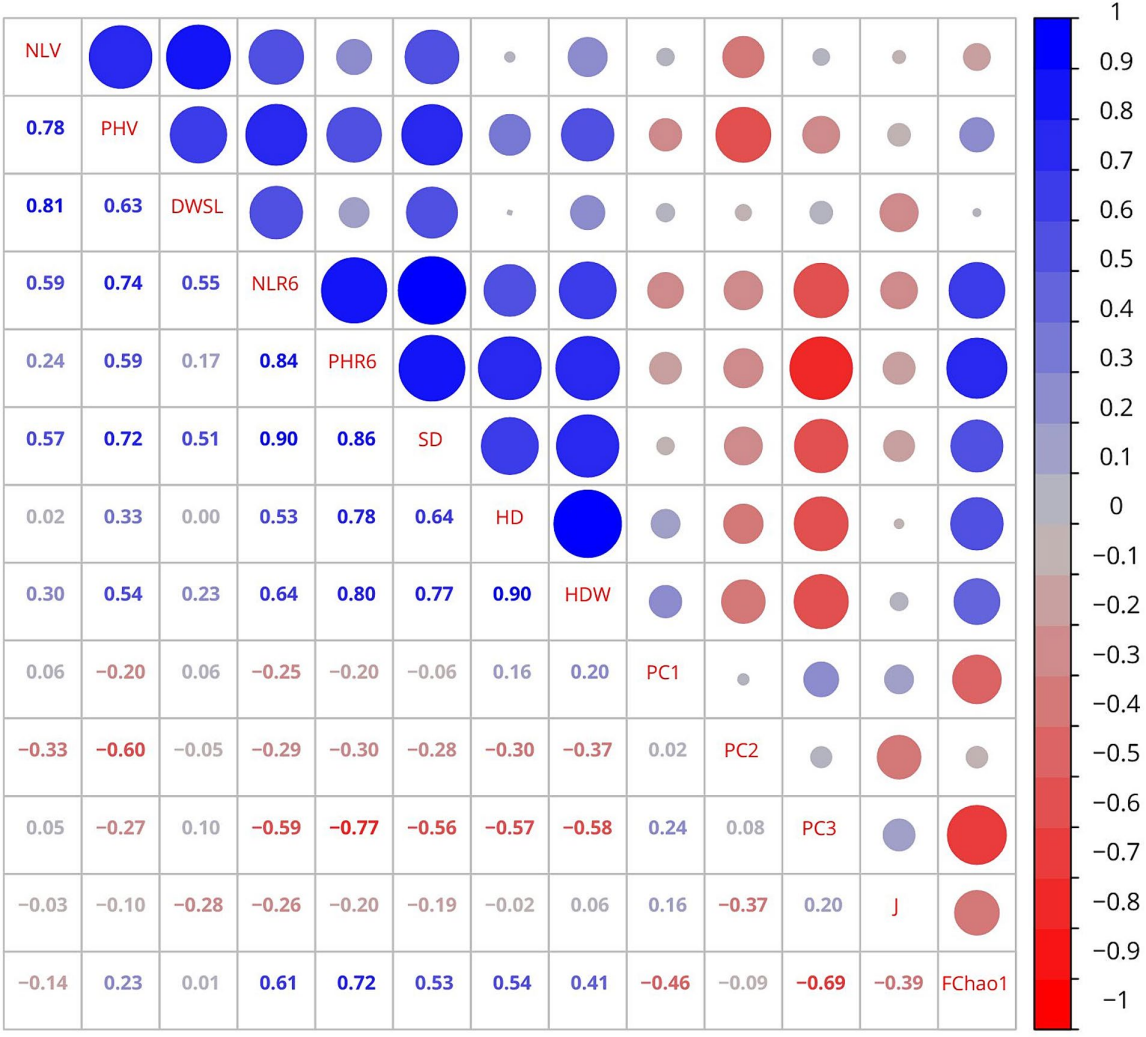
Heatmap depicting the matrix of Pearson’s correlation coefficients among the principal components (PC1, PC2, and PC3), Pielou’s evenness index (J), Chao-1 for fungi (FChao1), and sunflower growth parameters. NLV, number of leaves in vegetative; PHV, height in vegetative; DWSL, dry weight of stems and leaves; PHR6, height in R6; NLR6, number of leaves in R6; SD, stem diameter; HD, head diameter; HDW, head dry weight. The circle size and intensity of colors show the absolute value of Pearson’s correlation, while color represents the sign of the correlations (red, for negative, and blue for positive correlations).

A heatmap in Figure 4 shows Pearson’s correlation matrix, reporting the coefficients among the bacterial PC scores (PC1, PC2, and PC3), Pielou’s evenness index (J) for bacteria, Chao-1 for fungi (FChao1), and sunflower growth parameters (NLV: number of leaves in vegetative, PHV: plant height in vegetative, DWSL: dry weight of stems and leaves, PHR6: plant height in R6, NLR6: number of leaves in R6, SD: stem diameter, HD: head diameter, HDW: head dry weight). Overall, we found six very strong (>|0.8|), sixteen strong (|0.6–0.8|), and fifteen moderate (|0.4–0.6|) associations of statistical significance (*p* < 0.05). Thus, PC1 had a moderate negative association with FChao1. PC2 was associated moderately and negatively with plant height (PHV) and Pielou’s evenness index (J). Bacterial PC3 showed a very strong positive correlation with plant height (PHR6) and strong correlation with number of leaves (NLR6), stem diameter, head diameter, head dry weight, and FChao1. Also, PC3 was moderately and positively correlated with plant height (PHV).

Among the sunflower growth parameters examined, number of leaves in vegetative had a very strong positive association with dry weight of stems and leaves, strong positive correlations with plant height (PHV), and moderate with number of leaves (NLR6) and stem diameter. Plant height (PHV) was strongly and positively correlated with dry weight of stems and leaves, number of leaves (NLR6), and stem diameter and moderately and positively associated with plant height in R6 and head dry weight. Dry weight of stems and leaves had a moderate positive association with number of leaves (NLR6) and stem diameter. Number of leaves (NLR6) had a very strong positive association with plant height (PHR6) and stem diameter, strong with head dry weight and FChao1, and moderate with head diameter. Plant height (PHR6) showed a very strong positive correlation with stem diameter, strong with head diameter, head dry weight, and FChao1. Stem diameter was strongly and positively correlated with head diameter and head dry weight, and moderate with FChao1. Head diameter had a very strong positive association with head dry weight and moderate with FChao1.

## 4 Discussion

Previous research has underscored the importance of key growth parameters, including plant height, head diameter, and stem diameter, as critical determinants of grain yield in sunflower (Marinković, 1992; Kaya et al., 2007, 2009). This study was predicated on the hypothesis that the method of winter cover crop (CC) suppression—either chemical (glyphosate) or mechanical (rolling)—would significantly influence the growth and development of the subsequent sunflower crop within a rotational cycle. Our results support this hypothesis, revealing that both chemical and mechanical suppression of oats significantly affect sunflower’s vegetative and reproductive growth parameters.

Notably, sunflowers cultivated after chemically suppressed winter CC or maintained in fallow conditions (CT) exhibited enhanced growth compared to those following mechanical suppression. This distinction suggests that the mechanical method, particularly rolling, may not effectively manage oats and weeds, leading to increased competition for water, light, and nutrients. The observed limitations of rolling, particularly at the Zadoks 55 stage, for effective management of oats and weeds, aligns with Mirsky et al. (2009), who advocate for suppression at the later Zadoks 61 stage to optimize water management and weed suppression. However, literature on the impact of cover crop suppression methods on crop yields remains divided, with studies like Baigorria et al. (2019) and Antichi et al. (2022) reporting negligible effects on soybean and sunflower yields, highlighting the need for further exploration into optimizing mechanical suppression techniques.

What is interesting however, is that the sunflowers grown after a chemical fallow period with P fertilization (CT) had lower growth metrics compared to those following chemical oats suppression. This observation is congruent with findings from Tesfamariam et al. (2009) and Anza et al. (2016), which have documented potential adverse effects of glyphosate-based weeds control on primary crops and suggests that cover crops residues might mitigate such impacts.

Despite initial expectations, the use of starter P fertilization did not markedly alter growth outcomes across different chemical suppression treatments. This is consistent with the low mobility of phosphate ions in soil and the low doses of starter P applied as a granular formulation, close to the seeds, potentially minimizing glyphosate’s deleterious interactions within the soil environment, in contrast to Bott et al. (2011) who reported negative effects of liquid phosphorus fertilizers.

Diversity indices, both alpha and beta, serve as critical measures for assessing changes in soil microbial communities resulting from agricultural practices (Kim et al., 2022; Kodadinne Narayana et al., 2022). Soil microbial diversity plays a key role in maintaining numerous ecosystem functions and is positively correlated with plant biomass (Duchene et al., 2017; Chen et al., 2020). Coinciding with Morales et al. (2022), our study did not detect significant changes in most bacterial alpha diversity indexes across treatments. However, an increase in Pielou’s evenness index for sunflowers cultivated after winter CC suppression, regardless of the suppression method, suggests a more balanced and diverse bacterial community, akin to observations by Li et al. (2023) under a rye (*Secale cereale* L.) cover crop system.

Amplicon sequencing analysis did not reveal significant beta diversity changes in the bacterial community of the sunflower rhizosphere, mirroring findings from Morales et al. (2022) and Ouverson et al. (2022). Despite the lack of significant structural changes, treatment effects on community composition were evident, underscoring the sensitivity of compositional metrics over beta diversity in capturing microbial response to agricultural interventions.

This study underscores the effect of winter CC management on fungal diversity within the sunflower rhizosphere, especially when oats suppression is followed by P fertilization at planting. The observed increase in fungal richness, as indicated by the Chao-1 index, suggests beneficial alterations to the soil habitat due to the presence of cover crop to host more diverse fungal taxa, supporting the notion that cover crop can enrich soil biodiversity and function (Jousset et al., 2017; Yu et al., 2022).

The positive correlation between enhanced fungal richness and critical sunflower growth metrics highlights the potential benefits of a diverse fungal community for plant productivity, a finding that echoes the work of Zhang et al. (2022) on the role of fungal diversity in plant growth. This relationship suggests that integrated management practices, incorporating both winter CC and appropriate nutrient management, can significantly influence plant growth outcomes by fostering beneficial soil microbial conditions.

However, the stability observed in the overall fungal community structure across treatments points to the resilience and adaptability of soil fungal populations to sunflowers management practices. This adaptability underscores the complex interplay between soil management, microbial communities, and plant health, highlighting the need for further research to delve into the specific fungal taxa driving these benefits. Optimizing winter CC and nutrient management to leverage these soil microbial benefits could play a crucial role in advancing sustainable agricultural practices (Kim et al., 2022; Kodadinne Narayana et al., 2022).

The identification of specific bacterial and fungal genera that respond positively or negatively to agricultural treatments is crucial for understanding how these practices influence the microbial ecology of the rhizosphere and, by extension, plant health and productivity. In this study, amplicon sequencing revealed 16 bacterial genera that were significantly impacted by the applied treatments, with seven of these identified as potential plant growth-promoting rhizobacteria (PGPR). Many types of PGPR inhabit the plant rhizosphere, where they can influence plant growth and health directly or indirectly by suppressing pathogens, synthesizing phytohormones, fixing nitrogen, solubilizing phosphate, reducing heavy metals, improving plant tolerance to biotic and abiotic stress, etc. (Chandran et al., 2021; Mohanty et al., 2021).

Conversely, our findings regarding the fungal community contrast with those of the bacterial community, as fungal taxa did not show a clear response to the sunflower management treatments. This could be attributed to the dynamic and complex nature of fungal communities, which may require more pronounced environmental changes to elicit a detectable response. However, the role of fungi in plant health, particularly through mycorrhizal associations and pathogen suppression, remains undeniable (Rosner et al., 2018). The lack of significant treatment effects on fungal taxa suggests a resilience in the fungal community to the specific sunflowers management practices, contrasting with findings from Morales et al. (2022) that emphasize the potential for winter CC to act as a green bridge, carrying over saprophytic or pathogenic fungi to subsequent crops. Other factors not explored in our study, such as climate, soil type, or spatial distribution, may be more influential drivers of the fungal community richness and composition (Tedersoo et al., 2014).

Among the bacterial genera identified, *Aeromicrobium*, *Gaiella*, and *Niastella* were highlighted for their positive response across most treatments, excluding rolling with P. These genera have been associated with the promotion of plant growth and soil health in various studies (Oberholster et al., 2018; Cordero et al., 2020; Li et al., 2020). For example, *Aeromicrobium* has been implicated in the production of erythromycin, a macrolide antibiotic that may protect plants against soil-borne pathogens (Miller et al., 1991). Likewise, genera like *Stenotrophomonas* and *Novosphingobium*, favored in the rolling with P treatment, have been isolated from diverse plant species’ rhizospheres and are known to possess PGPR traits (Wolf et al., 2002; Zhang et al., 2016).

In our study, three bacterial genera—*Hymenobacter*, *Pseudarthrobacter*, and *Pelomicrobium*—showed a decreased presence in the rhizosphere of sunflower grown after chemical suppression of the winter CC, whereas *Sandaracinus* and *Chryseolinea* demonstrated a positive response to these treatments. *Hymenobacter* and *Pseudarthrobacter*, recognized for their plant growth-promoting capabilities, enhance germination rates and overall plant vigor (Dimitrijević et al., 2018; Issifu et al., 2022; Kabir et al., 2023). This suggests that while chemical suppression of the winter CC can negatively impact beneficial bacteria, such as those producing siderophores (Kumar et al., 2019), its overall effect on sunflower growth may be mitigated by the redundancy within the bacterial community. However, the reliance of these sunflower management practices (SMP) on chemical suppression poses potential long-term risks to the microbial diversity of the sunflower rhizosphere, necessitating research into optimizing mechanical suppression methods to minimize glyphosate use and its impact on soil microbiomes.

The genera *Sandaracinus* and *Chryseolinea*, which could act as biocontrol agents against soil-borne pathogens like *Fusarium* (Ou et al., 2019; Shrivastava and Sharma, 2021), were more prevalent in management practices that relied on chemical suppression. Their colonization in the sunflower rhizosphere might play a crucial role in disease management. Additionally, certain identified indicator bacteria—such as *Lentzea*, *Aeromicrobium*, *Thermanaerothrix*, and *Gemmatirosa*—have been linked with beneficial effects on plant health and productivity, from enhancing nutrient uptake to providing protection against pathogens (Miller et al., 1991; Wu et al., 2019; Li et al., 2020; Peng et al., 2022).

These findings underline the complex interactions between sunflower growth and rhizosphere microbial composition following winter CC suppression, whether through chemical or mechanical means. The identified bioindicators point toward the potential of tailored agricultural practices to not only influence crop yield positively but also support soil microbial diversity and function. Future agricultural strategies should aim to harness these interactions for the development of sustainable cultivation practices that balance productivity with ecological health.

## 5 Conclusion

This research highlights that incorporating a cover crop of oats into the traditional sunflowers crop rotations offers potential advantages for both the soil microbiota and crop productivity over conventional chemical fallow practices. We reveal that there are significant effects of using oats as a winter cover crop on the subsequent sunflower crop, demonstrating how the choice of suppression method—mechanical rolling versus chemical glyphosate application—distinctly influences plant growth and rhizosphere community composition.

Our findings show that while rolling can negatively impact sunflower growth, sunflowers cultivated following chemically suppressed oats exhibit improved performance on several reproductive parameters compared to those grown after a traditional chemical fallow without cover crops. However, glyphosate application to suppress the growth of oats before planting the cash crop was found to decrease the abundance of beneficial soil bacteria, whereas mechanical rolling appeared to preserve or even enhance their presence.

The study highlights the need for strategic adjustments to current winter cover crop management practices to mitigate the reliance on herbicides. Strategies could include optimizing mechanical suppression techniques to improve their effectiveness, selecting winter cover crop species that are amenable to both suppression methods, and exploring the synergistic use of mechanical rolling and herbicides application. Such approaches aim to reduce herbicide impacts on beneficial soil microbiota while ensuring adequate weed control and supporting sunflower growth, as well as enhancing nutrient availability and water retention.

The complexity unveiled by this study calls for a nuanced understanding of sustainable agricultural practices, advocating for a holistic approach that harmonizes crop performance objectives with rhizosphere microbial composition. Future research should help elucidate the underlying mechanisms through which winter cover crop management practices affect rhizosphere microbial communities and plant development. Ultimately, the goal is to devise practical, environmentally friendly strategies that optimize the benefits of microbial diversity for enhancing the sustainability and productivity of agricultural systems.

## Data availability statement

The datasets presented in this study can be found in online repositories. The names of the repository/repositories and accession number(s) can be found at: https://www.ncbi.nlm.nih.gov/sra, PRJNA1034035.

## Author contributions

MM: Data curation, Formal analysis, Investigation, Visualization, Writing – original draft, Writing – review & editing. MA: Data curation, Formal analysis, Investigation, Writing – review & editing. JB: Investigation, Writing – review & editing. GI: Investigation, Writing – review & editing. MV: Conceptualization, Formal analysis, Funding acquisition, Project administration, Resources, Supervision, Writing – review & editing. MZ: Conceptualization, Funding acquisition, Investigation, Project administration, Resources, Supervision, Writing – review & editing.
