# Supplementary material for: Winter cover crop suppression methods influence on sunflower growth and rhizosphere communities
Source: Front Microbiol. 2024 Jun 26;15:1405842. doi: 10.3389/fmicb.2024.1405842 (PMC11238176; doi:10.3389/fmicb.2024.1405842)
Supplement: Supplementary file 1 [file Data_Sheet_1.docx]

Supplementary Material

1. Supplementary Figures and Tables

**Supplementary Table S1.** Supplementary irrigation.

| Year | Months | Irrigation water (mm) |
| --- | --- | --- |
| 2018 | November | 6 |
| 2018 | December | 6 |
| 2019 | November | 12 |
| 2019 | December | 60 |

**Supplementary Table S2.** The primer sets used for amplification.

| Target group | Primers | Sequence 5’→3’ |
| --- | --- | --- |
| Bacteria | 515F  806R | GTGCCAGCMGCCGCGGTAA  GGACTACVSGGGTATCTAAT |
| Fungi | 7F  4R | GTGARTCATCGAATCTTTG  TCCTCCGCTTATTGATATGC |

**Supplementary Table S3**. Comparison of metagenomics data between treatments using the Benjamini and Hochberg (“FDR”) correction.

| Comparison | PERMANOVA (Generalized UniFrac distance) |
| --- | --- |
|  | P value with corrections |
| DQ vs CT | 0.16 |
| DQ/P vs CT | 0.16 |
| R vs CT | 0.24 |
| R/P vs CT | 0.16 |
| DQ/P vs DQ | 0.53 |
| R vs DQ | 0.16 |
| R vs DQ/P | 0.24 |
| R/P vs DQ | 0.22 |
| R/P vs DQ/P | 0.22 |
| R/P vs R | 0.52 |

Treatments: CT, control treatment; DQ/P, sunflower after winter CC chemical suppression with P fertilization at sowing; DQ, sunflower after winter CC chemical suppression without P at sowing; R/P, sunflower after winter CC rolling with P fertilization at sowing; R, sunflower after winter CC rolling without P at sowing.

**Supplementary Table S4.** Principal component analysis of bacteria genera showing the eigenvector, the eigenvalue, and the cumulative proportion of the dataset variability explained by each of the eight principal components (PCs) extracted. Genera with loadings >|0.45| are bolded.

|  |  | PC1 | PC2 | PC3 | PC4 | PC5 | PC6 | PC7 | PC8 |
| --- | --- | --- | --- | --- | --- | --- | --- | --- | --- |
| Eigenvalue |  | 4.53 | 3.08 | 2.76 | 2.19 | 1.73 | 1.48 | 1.34 | 1.32 |
| Proportion |  | 0.17 | 0.12 | 0.11 | 0.08 | 0.07 | 0.06 | 0.05 | 0.05 |
| Cum. proportion |  | 0.17 | 0.29 | 0.40 | 0.48 | 0.55 | 0.61 | 0.66 | 0.71 |
|  |  | Component correlation scores | | | | | | |  |
| *Aciditerrimonas* |  | **0.71** | 0.30 | −0.18 | −0.01 | 0.01 | −0.33 | −0.02 | 0.05 |
| *Aeromicrobium* |  | **0.47** | 0.22 | **0.60** | −0.15 | 0.04 | 0.13 | −0.04 | 0.34 |
| *Agromyces* |  | 0.17 | 0.34 | 0.25 | **−0.61** | 0.16 | −0.31 | −0.03 | 0.23 |
| *Arma_gp5* |  | 0.10 | −0.21 | −0.06 | **0.71** | 0.34 | −0.24 | 0.24 | 0.06 |
| *Chitinispirillum* |  | 0.35 | −0.09 | −0.20 | 0.14 | −0.02 | −0.11 | −0.02 | **0.51** |
| *Chryseolinea* |  | 0.43 | **−0.80** | 0.15 | −0.13 | −0.15 | −0.01 | 0.15 | −0.10 |
| *Denitratisoma* |  | 0.32 | 0.23 | −0.25 | **0.52** | −0.24 | 0.13 | **−0.48** | 0.20 |
| *Gaiella* |  | **0.72** | 0.43 | −0.22 | 0.02 | 0.23 | −0.01 | 0.07 | 0.16 |
| *Gemmatirosa* |  | **0.50** | 0.21 | **−0.49** | 0.20 | 0.05 | 0.22 | 0.27 | 0.08 |
| *Hymenobacter* |  | −0.42 | **0.60** | 0.17 | 0.08 | 0.24 | −0.16 | 0.16 | 0.03 |
| *Jatrophihabitans* |  | −0.07 | 0.38 | -0.42 | −0.37 | 0.18 | −0.28 | 0.05 | −0.15 |
| *Lentzea* |  | 0.27 | 0.18 | **0.49** | 0.10 | −0.38 | −0.05 | 0.40 | −0.08 |
| *Limnoglobus* |  | **0.47** | 0.17 | 0.30 | −0.06 | 0.35 | 0.20 | −0.18 | **−0.46** |
| *Marmoricola* |  | 0.40 | −0.03 | 0.19 | −0.36 | 0.32 | 0.15 | −0.32 | 0.20 |
| *Mesorhizobium* |  | 0.27 | −0.13 | −0.40 | 0.01 | 0.42 | 0.23 | 0.38 | 0.13 |
| *Mucilaginibacter* |  | −0.13 | −0.35 | −0.33 | −0.02 | 0.15 | **−0.47** | −0.12 | 0.29 |
| *Niastella* |  | **0.59** | −0.29 | 0.19 | −0.22 | −0.35 | 0.19 | 0.22 | 0.12 |
| *Novosphingobium* |  | **−0.48** | −0.08 | 0.15 | 0.33 | 0.15 | 0.41 | 0.11 | 0.37 |
| *Ornatilinea* |  | 0.44 | −0.43 | 0.27 | 0.24 | 0.16 | −0.27 | 0.12 | −0.24 |
| *Pantoea* |  | −0.23 | 0.00 | 0.42 | 0.31 | **0.46** | 0.07 | −0.36 | −0.11 |
| *Pelomicrobium* |  | 0.00 | **0.50** | −0.38 | −0.13 | 0.05 | 0.27 | 0.31 | −0.28 |
| *Pseudarthrobacter* |  | 0.35 | **0.51** | **0.53** | 0.20 | −0.19 | 0.06 | 0.06 | 0.13 |
| *Sandaracinus* |  | 0.25 | **−0.58** | −0.22 | −0.43 | 0.20 | 0.27 | −0.09 | 0.04 |
| *Stenotrophomonas* |  | **−0.57** | 0.03 | 0.06 | −0.23 | 0.21 | 0.40 | 0.14 | 0.22 |
| *Thermanaerothrix* |  | 0.12 | 0.18 | **−0.50** | 0.02 | **−0.46** | 0.22 | −0.36 | −0.11 |
| *Zavarzinella* |  | **0.73** | −0.04 | −0.04 | 0.21 | 0.29 | 0.19 | −0.13 | −0.22 |
|  | *df* | P-value | | | | | | |  |
| Treatments | 4 | 0.012 | <0.0001 | 0.030 | 0.145 | 0.086 | 0.409 | 0.284 | 0.262 |

**Supplementary Table S5**. Mean values (mean), standard errors of the mean (SEM), probability values (p-values), number of observations (n), and degrees of freedom (df) associated with the analysis of variance (ANOVA) results for the effect of the treatments on each fungi genera.

|  |  | Fungi | | | | | | | |
| --- | --- | --- | --- | --- | --- | --- | --- | --- | --- |
| Treatments^1^ |  | *Alternaria* | | *Arthrinium* | | *Atradidymella* | | *Auricularia* | |
|  | n | Mean | SEM | Mean | SEM | Mean | SEM | Mean | SEM |
| CT | 7 | 3.88 | 1.20 | −1.97 | 1.64 | 3.55 | 1.60 | −0.66 | 2.06 |
| DQ | 7 | 2.67 | 1.20 | −1.94 | 1.64 | −1.62 | 1.60 | −0.75 | 2.06 |
| DQ/P | 7 | 3.79 | 1.20 | −0.34 | 1.64 | −0.92 | 1.60 | −0.23 | 2.06 |
| R | 7 | 2.02 | 1.20 | −3.29 | 1.64 | −2.06 | 1.60 | 2.87 | 2.06 |
| R/P | 6 | 4.26 | 1.28 | −2.81 | 1.68 | −2.42 | 1.66 | 3.16 | 2.12 |
|  | df | P-value | | | | | | | |
|  | 4 | 0.627 | | 0.436 | | 0.157 | | 0.531 | |

^1^ Treatments: CT, control treatment; DQ/P, sunflower after winter CC chemical suppression with P fertilization at sowing; DQ, sunflower after winter CC chemical suppression without P at sowing; R/P, sunflower after winter CC rolling with P fertilization at sowing; R, sunflower after winter CC rolling without P at sowing.


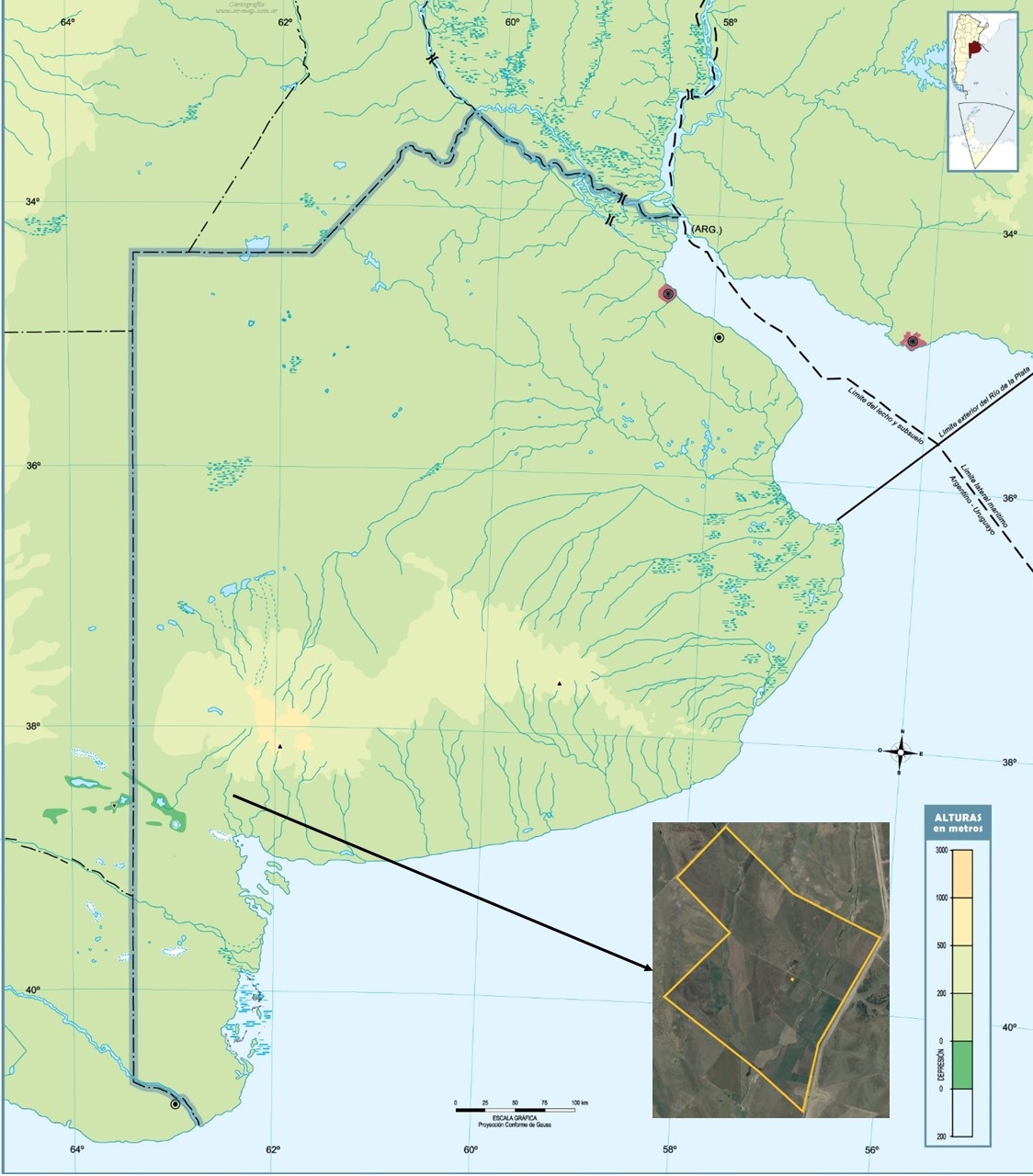


**Supplementary Figure S1.** Location of the experimental site in Bahía Blanca, Buenos Aires Province, Argentina.


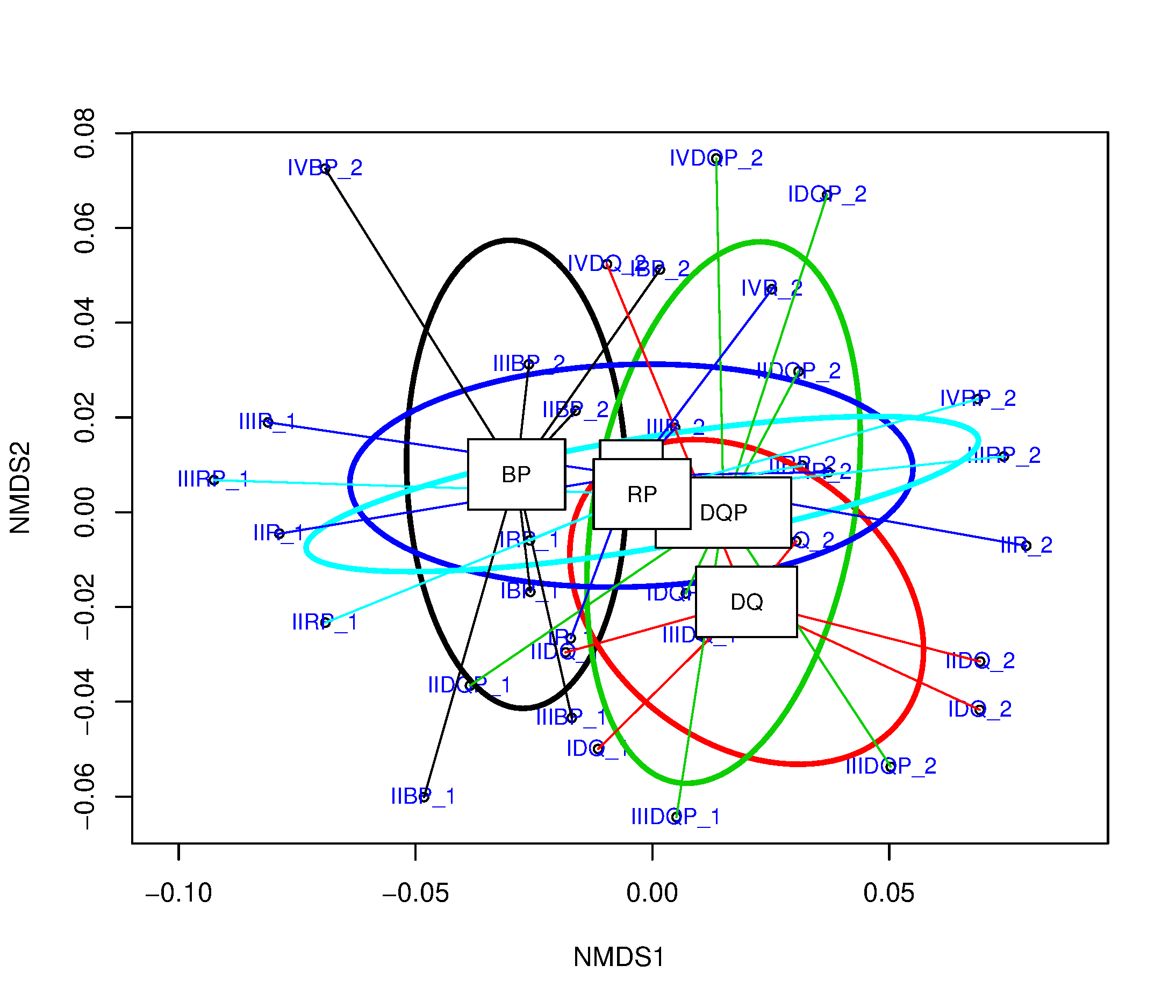

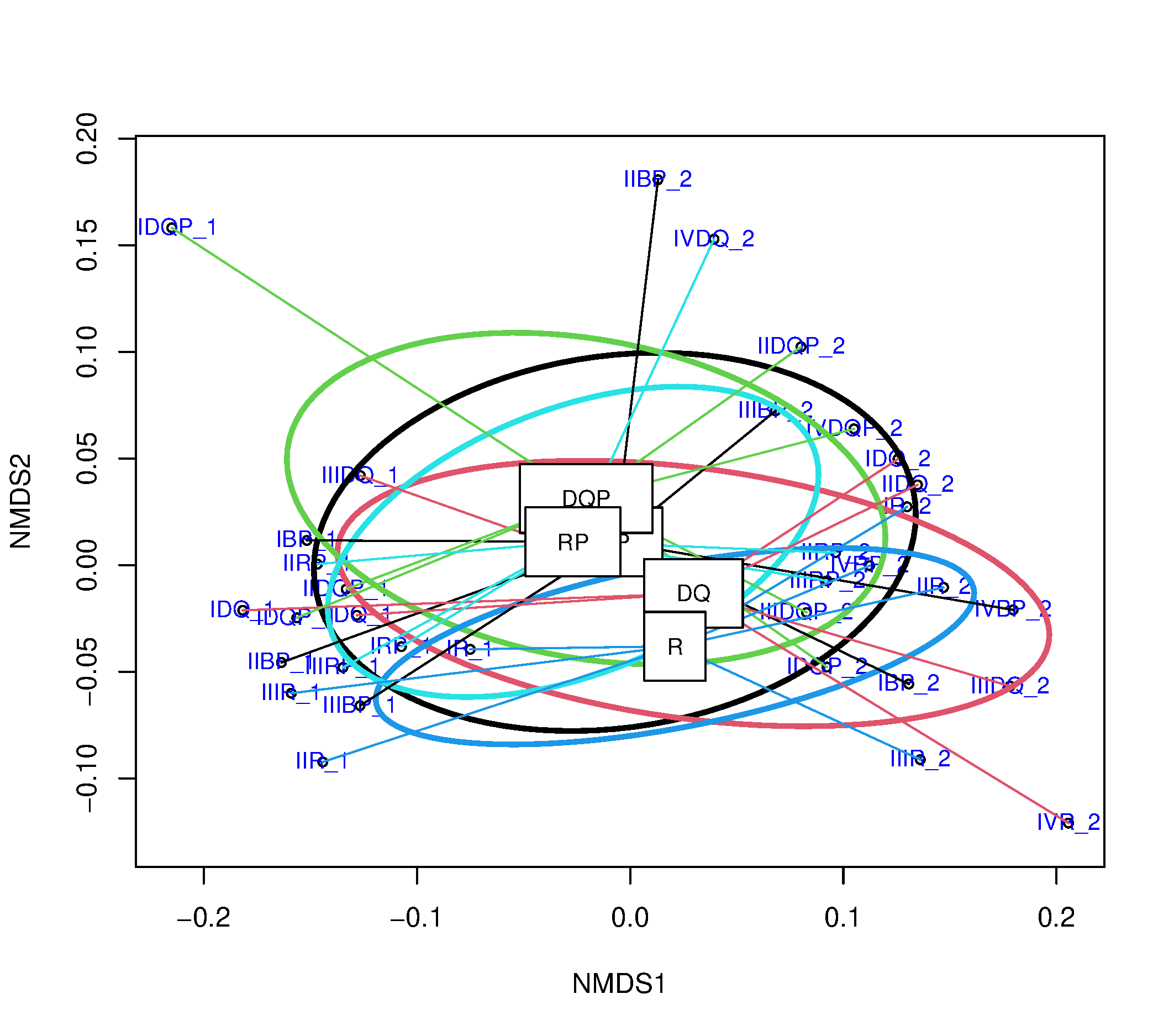


1. **(B)**

**Supplementary Figure S2.** Non-metric multidimensional scaling of Bacteria (Stress = 0.16) (A) and Fungi (Stress = 0.14) (B) using generalized UniFrac distance. The labels "DQ" indicate sunflower after winter CC chemical suppression without P at sowing, "DQP" denotes sunflower after winter CC chemical suppression with P fertilization at sowing, "R" sunflower after winter CC rolling without P at sowing, "RP" signifies sunflower after winter CC rolling with P fertilization at sowing, and "BP (=CT)" denotes control treatment. Roman numerals (I to IV) identify the replicates, with "1" indicating samples from 2018 and "2" indicating samples from 2019. Centroids are indicated by boxes. The standard error of "DQ", "DQP", "R", "RP", and "BP (=CT)" are indicated by red, green, blue, light blue, and black ellipses, respectively.
